# National‐scale distribution of protists associated with sorghum leaves and roots

**DOI:** 10.1111/1758-2229.70024

**Published:** 2024-10-01

**Authors:** Peng He, Anqi Sun, Xiaoyan Jiao, Peixin Ren, Fangfang Li, Bingxue Wu, Ji‐Zheng He, Hang‐Wei Hu

**Affiliations:** ^1^ Key Laboratory for Humid Subtropical Eco‐geographical Processes of the Ministry of Education, School of Geographical Sciences Fujian Normal University Fuzhou China; ^2^ Key Laboratory of Urban Environment and Health, Ningbo Urban Environment Observation and Research Station Institute of Urban Environment, Chinese Academy of Sciences Xiamen China; ^3^ College of Resources and Environment Shanxi Agricultural University Taiyuan China; ^4^ School of Agriculture, Food and Ecosystem Sciences, Faculty of Science The University of Melbourne Parkville Victoria Australia

## Abstract

Protists, as integral constituents of the plant microbiome, are posited to confer substantial benefits to plant health and performance. Despite their significance, protists have received considerably less attention compared to other constituents of the plant microbiome, such as bacteria and fungi. To investigate the diversity and community structure of protists in sorghum leaves and roots, we employed amplicon sequencing of the eukaryotic 18S rRNA gene in 563 leaf and root samples collected from 57 locations across China. We found significant differences in the diversity and community structure of protists in sorghum leaves and roots. The leaf was taxonomically dominated by Evosea, Cercozoa and Ciliophora, while the root was dominated by Endomyxa, Cercozoa and Oomycota. The functional taxa of protists exhibited notable differences between leaves and roots, with the former being predominantly occupied by consumers and the latter by parasites. The community composition of protists in the leaf was predominantly influenced by mean annual precipitation, whereas soil pH played a more significant role in the root. The present study identified the most abundant and distributed protists in sorghum leaves and roots and elucidated the underlying factors that govern their community structure. The present study offers a novel perspective on the factors that shape plant‐associated protist communities and their potential roles in enhancing the functionality of plant ecosystems.

## Graphical abstract

Significant differences were observed in the diversity and community composition of protists between sorghum leaves and roots. Leaf protist communities were primarily dominated by consumers, while root protists were predominantly parasites. The structure of the leaf protist community was largely influenced by mean annual precipitation, whereas soil pH was the primary driver of changes in the root protist community.

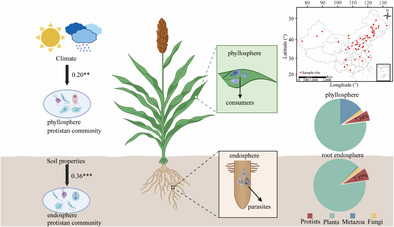

## INTRODUCTION

Distinct plant compartments, including the phyllosphere, rhizosphere and endosphere, offer diverse habitats for a wide array of microorganisms such as bacteria, fungi, protists, archaea (Beckers et al., 2017; Hassani et al., 2018; Taerum et al., 2023; Taffner et al., 2018; Trivedi et al., 2020). Microbiomes deliver both direct and indirect advantages to host plants, which include the facilitation of growth, the enhancement of nutrient turnover and the fortification of defences against pathogenic organisms (Gouda et al., 2018; Hubbard et al., 2019; Pieterse et al., 2014; Trivedi et al., 2016). Owing to the distinct morphologies of plant roots and leaves, coupled with their varying degrees of interaction with soil and atmosphere, there is a divergence in the microbial community composition influenced by edaphic and climatic variables (Fitzpatrick et al., 2020; Sohrabi et al., 2023; Zhu et al., 2022). In the exploration of microbiomes across various plant compartments, bacterial and fungal taxa predominantly constitute the primary subjects of previous research (De Souza et al., 2016; Fitzpatrick et al., 2018; Sun, Jiao, Chen, Wu, et al., 2021). Despite the fact that protists serve as integral constituents of the plant microbiome, our knowledge regarding the community dynamics of plant‐associated protists and their functions remains limited (Nguyen et al., 2023; Trivedi et al., 2020).

Protists include all eukaryotic organisms except land plants, animals and fungi (Geisen et al., 2018). Protists are abundant and widespread unicellular eukaryotes that span the entire eukaryotic tree of life (Adl et al., 2019), exhibit a broad distribution and perform critical ecological functions (Gao et al., 2019; Geisen et al., 2018; Huang et al., 2021). Protists occupy a range of trophic functional groups, including consumers, photobionts and pathogens of most plants and animals (Geisen et al., 2018). As consumers, protists play an important role in nutrient cycling, plant nutrient uptake and pathogen control (Nguyen et al., 2021). For example, previous studies reported that phyllosphere and rhizosphere consumer protists, through trophic interactions, exert regulatory control over the composition of bacterial and fungal communities, which subsequently contributes to the facilitation of plant growth (Guo et al., 2021; Ren et al., 2023; Sun, Jiao, Chen, Trivedi, et al., 2021; Xiong et al., 2020). Protists can not only feed on other organisms, but also parasitise them (Geisen et al., 2018). Oomycetes, a group of parasitic protists prevalent in plant roots, are ubiquitous plant pathogens capable of causing devastating crop diseases and widespread mortality (Larousse & Galiana, 2017; Mahé et al., 2017; Schwelm et al., 2018). Phototrophs increase oxygen levels through photosynthesis, thereby promoting plant growth and participating in biogeochemical processes (Geisen et al., 2018; Jassey et al., 2022; Lee & Ryu, 2021).

Protists exert a significant impact on plant growth processes, yet there is a limited understanding of the distribution and ecological preferences of plant‐associated protists across broad spatial scales. In the plant microbiome, where bacteria and fungi are still the main focus of research, plant‐associated protists and their functions for the plant host are largely underestimated (Gao et al., 2019; Trivedi et al., 2020). Majority of previous studies predominantly focused on the protists inhabiting the rhizosphere or soil environments (Guo et al., 2024; Nguyen et al., 2021; Oliverio et al., 2020; Sun, Jiao, Chen, Trivedi, et al., 2021), and studies of plant‐associated protists have been confined to a relatively narrow spectrum of environmental conditions at a regional scale (Ceja‐Navarro et al., 2021; Sapp et al., 2018). Therefore, it is imperative to elucidate the diversity and community composition of protists within distinct plant compartments, and the factors shaping their biogeographic distribution patterns at larger spatial scales. Bridging this knowledge gap will enhance our capabilities to predict the changes in plant‐associated protist communities and their ecological functions under the future scenarios of climate change.

This study aimed to characterize the diversity and community structure of protists in the leaf and root of sorghum and assess the relative influence of soil properties and climatic factors in shaping the distribution of sorghum‐associated protist communities at a national scale. Sorghum is remarkably versatile, serving as both a valuable food crop and a sustainable feedstock for bioenergy production (Mullet et al., 2014). Sorghum is widely grown for grain, feed, fibre and fuel and is widely used in winemaking (Liu et al., 2023). In this study, we investigated the protist communities in the leaf and root compartments of 563 sorghum samples, employing amplicon sequencing of the 18S rRNA gene. We aimed to address the following questions: (i) Is there a disparity in the diversity and community compositions of protists between the leaf and root of sorghum? (ii) Which protists constitute the predominant taxa within the leaf and root compartments? (iii) What climatic and edaphic factors regulate the large‐scale distribution of the protist communities in these plant‐associated compartments?

## EXPERIMENTAL PROCEDURES

### Site description and sample collection

**FIGURE 1:**
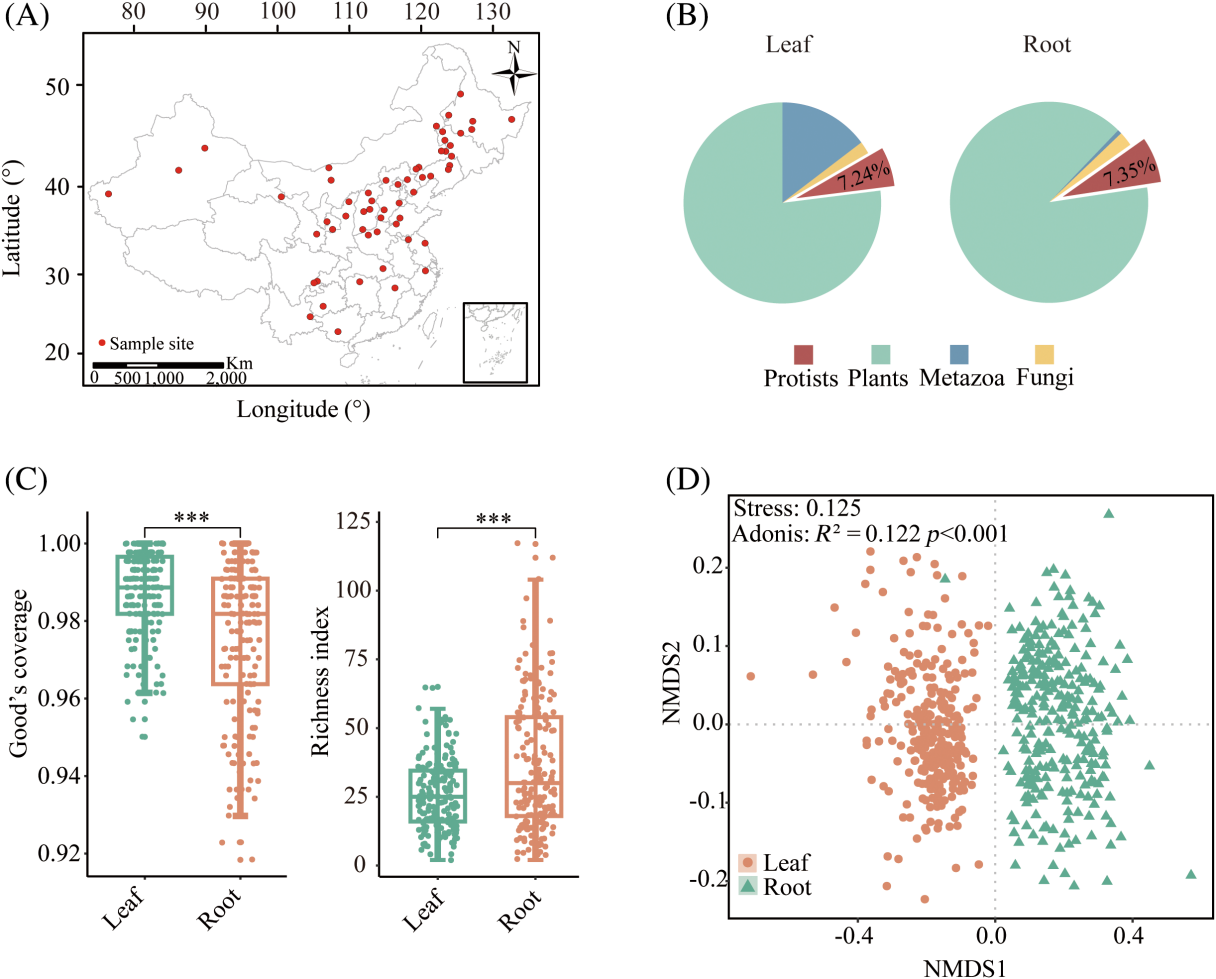
A map showing the location of 57 sampling sites at large spatial scales (A). Community compositions of eukaryotes in sorghum leaf and root (B). The Goods coverage and Richness diversity for the protistan communities across different sorghum‐associated compartments (***, *p* < 0.001; Wilcoxon rank sum test) (C). Non‐metric multidimensional scaling (NMDS) showing the distribution patterns of protists in leaf and root.

Our survey regions spanned from 22.85° N to 49.18° N and from 76.38° E to 132.38° E, covering the majority of China (Figure 1A). During the peak maturity phase of sorghum (July–August 2021), we systematically collected leaf and root samples from 57 distinct experimental field sites (Table S1). In this sampling campaign, five replicate samples were collected at each site, resulting in a total of 563 samples, including 279 leaf and 284 root samples. Sterilized scissors were used to collect plant leaf samples at a consistent height (~60 cm above soil surface) from the sorghum, with five leaves collected from each plant. The sorghum plants were extracted from the soil, and the roots were cut using sterilized scissors and placed into a sterile bag. Plant leaf samples and their corresponding root samples were taken from the same sorghum plants. All plant and soil samples were shipped to the laboratory in ice boxes and stored at −80°C prior to DNA extraction.

### Soil physicochemical analyses and climatic factors

Soil samples were analysed for the following soil properties using standard laboratory methods: soil pH, soil electrical conductivity (EC), soil moisture content (SMC), total nitrogen (TN), total carbon (TC), total phosphorus (TP), dissolved organic carbon (DOC), dissolved organic nitrogen (DON), nitrate nitrogen, ammonium nitrogen and available phosphorus (AP) (Ma et al., 2016). We obtained climatic data from the WorldClim database based on latitude and longitude of the 57 experimental sites (Fick & Hijmans, 2017). The data included mean annual temperature (MAT, 0.69–22.06°C), mean annual precipitation (MAP, 62.00–1633.00 mm), precipitation of wettest month, precipitation of driest quarter, precipitation of qarmest quarter, mean temperature of wettest quarter, mean temperature of warmest quarter and max temperature of warmest month (https://www.worldclim.org; ~1 km resolution).

### DNA extraction and sequencing

To characterize the protist communities inhabiting sorghum leaves and roots, we employed the amplicon sequencing of the 18S rRNA gene (Oliverio et al., 2018). The leaf sample site involved randomly selecting crushed leaves, taking 5 g the sample, rinsing it with phosphate buffer saline and shaking at 180 rpm at room temperature in an incubator for 1 h after ultrasonic shaking. The washing solution was passed through sterilized medical gauze and a 0.22‐μm filter by a Vacuum Filtration System (MultiVac610‐MS‐T, Rocker, China). Sorghum leaf DNA was extracted through the filtrate obtained after filtration using the DNeasy PowerSoil DNA isolation kit (Qiagen, Hilden, Germany) (Sun, Jiao, Chen, Wu, et al., 2021). Sorghum root samples are processed by surface sterilization using a 30% hydrogen peroxide solution, followed by sequential rinses with sterile deionized water and 70% ethanol. After rinsing, excess moisture was removed from the sample surface using sterile absorbent filter paper. The root sample was first cut into small fragments and pulverized under cryogenic conditions using liquid nitrogen. Then 0.25 g of the sample was weighed and DNA extracted using the DNeasy PowerSoil DNA Isolation Kit (Qiagen, Hilden, Germany) (Sun, Jiao, Chen, Wu, et al., 2021). The quantity of the DNA sample was assessed using the NanoDrop 2000 spectrophotometer (Thermo Scientific, United States).

Amplification of the eukaryotic 18S rRNA gene was conducted using the primer set F‐TAR‐euk454FWD1 and R‐TAReukREV3 to evaluate the protist communities within the leaf and root (George et al., 2019). The PCR reaction mixture including 4 μL 5× Fast Pfu buffer, 2 μL 2.5 mM dNTPs, 0.8 μL each primer (5 μM), 0.4 μL Fast Pfu polymerase, 10 ng of template DNA and ddH2O to a final volume of 20 μL. PCR amplification cycling conditions were as follows: initial denaturation at 95°C for 3 min, followed by 27 cycles of denaturing at 95°C for 30 s, annealing at 55°C for 30 s and extension at 72°C for 45 s, and single extension at 72°C for 10 min. The PCR products were extracted from 2% agarose gel and purified using the PCR Clean‐Up Kit (YuHua, Shanghai, China) according to manufacturer's instructions and quantified using Qubit 4.0 (Thermo Fisher Scientific, USA).

Sequencing was conducted using the Illumina MiSeq PE300 platform (Illumina, CA, USA). The QIIME software was employed for the denoising of high‐quality sequences using the default parameters (Caporaso et al., 2010). The raw sequences were discarded if they contained ambiguous nucleotides, had a low (*Q* <20) quality score and were short in length (<100 bp). Amplicon sequence variants (ASVs) with 100% similarity were identified using UPARSE (Edgar, 2013). The taxonomic classification of ASVs was conducted using the Protist Ribosomal Reference Database (PR2, version 4.14.0) (Guillou et al., 2012). Any ASVs with a taxonomic assignment of ‘Fungi, Metazoa and Viridiplantae’ were removed (Oliverio et al., 2020). Amplicon sequencing of the eukaryotic 18S rRNA gene resulted in a total of 20,776,004 sequences across all plant samples.

### Statistical analyses

To explore the relationships between environmental factors and the protistan diversity and community composition, the following statistical analyses were carried out. The alpha diversity of protists was determined based on the standardized ASVs using the ‘vegan’ package in *R*. Significant difference in the protist richness between the leaf and root was evaluated using the Wilcoxon rank‐sum test, with a threshold for statistical significance established at *p* < 0.05. To explore the distribution patterns of protist communities within the leaf and root, non‐metric multidimensional scaling (NMDS) analysis was conducted based on the Bray–Curtis dissimilarity metrics. Permutational multivariate analysis of variance (PERMANOVA), with 999 permutations, was performed to determine the influence of various factors on community dissimilarity using the ‘vegan’ package. Given the high degree of correlation among certain climatic variables within this sample set, collinearity was mitigated by excluding a subset of variables with Pearson correlation coefficients *R* of 0.8 or greater.

To elucidate the correlations between protist richness and climatic and edaphic variables, an ordinary least squares (OLS) regression model was employed, using the ‘lm’ function in R. Mantel test was conducted to identify the determinants influencing alterations in protist community composition using the ‘vegan’ package. To measure the impact of edaphic and climatic factors on the structure of the protist community and protist functional groups, the random forest analysis was conducted using the ‘randomForest’ package (Liaw & Wiener, 2002). The association between climatic and edaphic factors and the relative abundance of various functional groups at the genus level was analysed using the Spearman's rank correlation coefficient. All data analysis was conducted in R 4.3.1 and visualized using the ‘ggplot2’ package.

Structural equation models (SEMs) were employed to elucidate the direct and indirect influences of spatial parameters (latitude), climatic variables (MAT, MAP) and soil characteristics (pH, EC, MSC, TN, TC, TP, DOC, DON, AP, NO_3_^−^‐N, NH_4_^+^‐N) on the richness and community composition of protists in the leaf and root. Within this model, the NMDS1 + NMDS2 axis denoted community composition, and edaphic factors were represented by first axes of Principal Component Analysis (PCA1). AMOS 26 Graphics (IBM, USA) was used for the construction of the model with the maximum likelihood estimation method. The chi‐square test (*p* > 0.05), the root mean square error of approximation (RMSEA) (≤0.08), and the goodness‐of‐fit index (GFI) (≥0.95) were used to confirm the model's validity.

## RESULTS

### Compositions of protistan communities across different plant compartments

We analysed differences in the diversity and composition of protistan communities in the leaf and root of sorghum. We identified 670,883 and 845,636 ASVs of protists in the leaf and root, respectively. The good's coverage of protists was 98.74% ± 1.19% and 98.19% ± 1.63% in the leaf and root, respectively, indicative of sufficient sequencing depth to describe the protistan community diversity (Figure 1C). In the leaf, protists accounted for 7.24% of the total 18S rRNA gene sequences, which were dominated by the Plant kingdom (70.77%), followed by fungi (21.36%) and Metazoa (0.62%) (Figure 1B). Within the root, the sequences were predominantly categorized as plants (89.64%), followed by protists (7.35%), Metazoa (2.32%) and fungi (0.70%) (Figure 1B). The richness of protists was significantly lower in the leaf than in root (Wilcox‐test, *p* < 0.05) (Figure 1C). The NMDS ordination demonstrated a significant shift in the structure of the protistan community between the leaf and root (PERMANOVA, *p* < 0.001) (Figure 1D).

**FIGURE 2:**
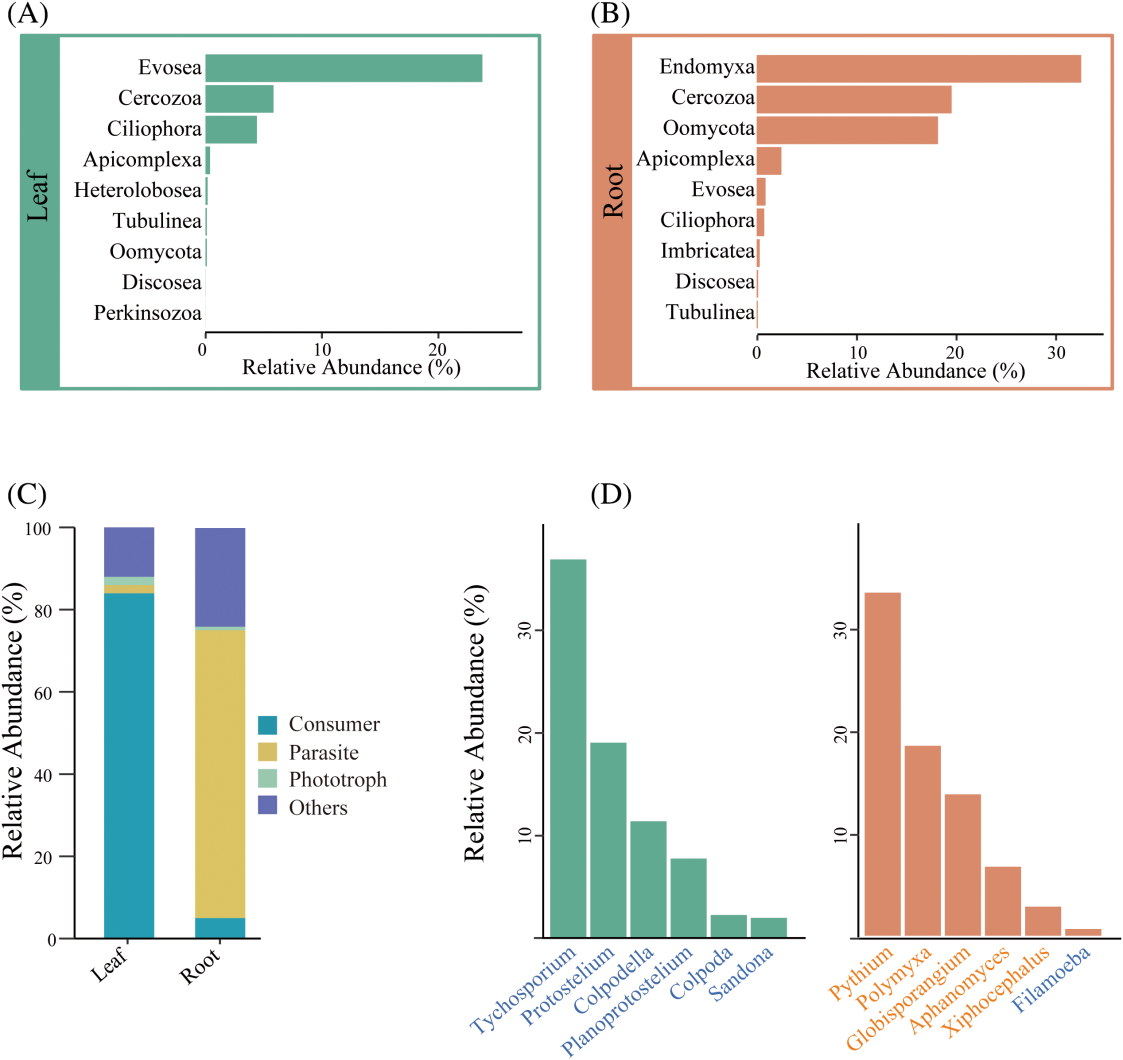
Changes in relative abundance of dominant protistan taxa (at the phylum level) in leaf (A) and root (B). The relative abundance of functional groups (C) and their dominant taxa (at the genus level) across different sorghum‐associated compartments (D).

The relative abundance of protists was taxonomically predominated by the phylum Evosea (23.77%) in the leaf, followed by Cercozoa (5.84%), Ciliophora (4.42%) and Apicomplexa (0.39%) (Figure 2A). Other less abundant protist phyla included Heterolobosea, Tubulinea, Oomycota, Discosea and Perkinsozoa. In the root, Endomyxa was the most dominant phylum, comprising 32.59% of the protist sequences, followed by Cercozoa (19.57%), Oomycota (18.20%) and Apicomplexa (2.48%). Meanwhile, less abundant Evosea, Ciliophora, Imbricatea, Discosea and Tubulinea existed in root endosphere (Figure 2B). Our analyses showed that the protist communities of sorghum leaves and roots varied among sampling sites (Figures S1 and S1). Surprisingly, 65.15% and 24.96% of the protists in the leaf and root samples, respectively, were unclassified.

This study further assigned the major protistan lineages (excluding unclassified) to their dominant mode of energy acquisition, including consumers, phototrophs and parasites and others. Leaf samples were dominated by protistan consumers (84.51%), compared with a dominance of parasites (70.01%) in root samples (Figure 2C). At the genus level, *Tychosporium* (37.25%), *Protostelium* (19.21%) and *Colpodella* (11.46%) dominated the leaf consumer protists (Figure 2D). The parasite protists were predominated by *Pythium* (30.31%) across root, followed by *Polymyxa* (16.78%) and *Globisporangium* (12.49%) (Figure 2D). Similarly, we found significant differences in functional taxa of protists across different sampling locations (Figures S2A and S2B).

### Drivers of the variation in protists in different compartments

**FIGURE 3:**
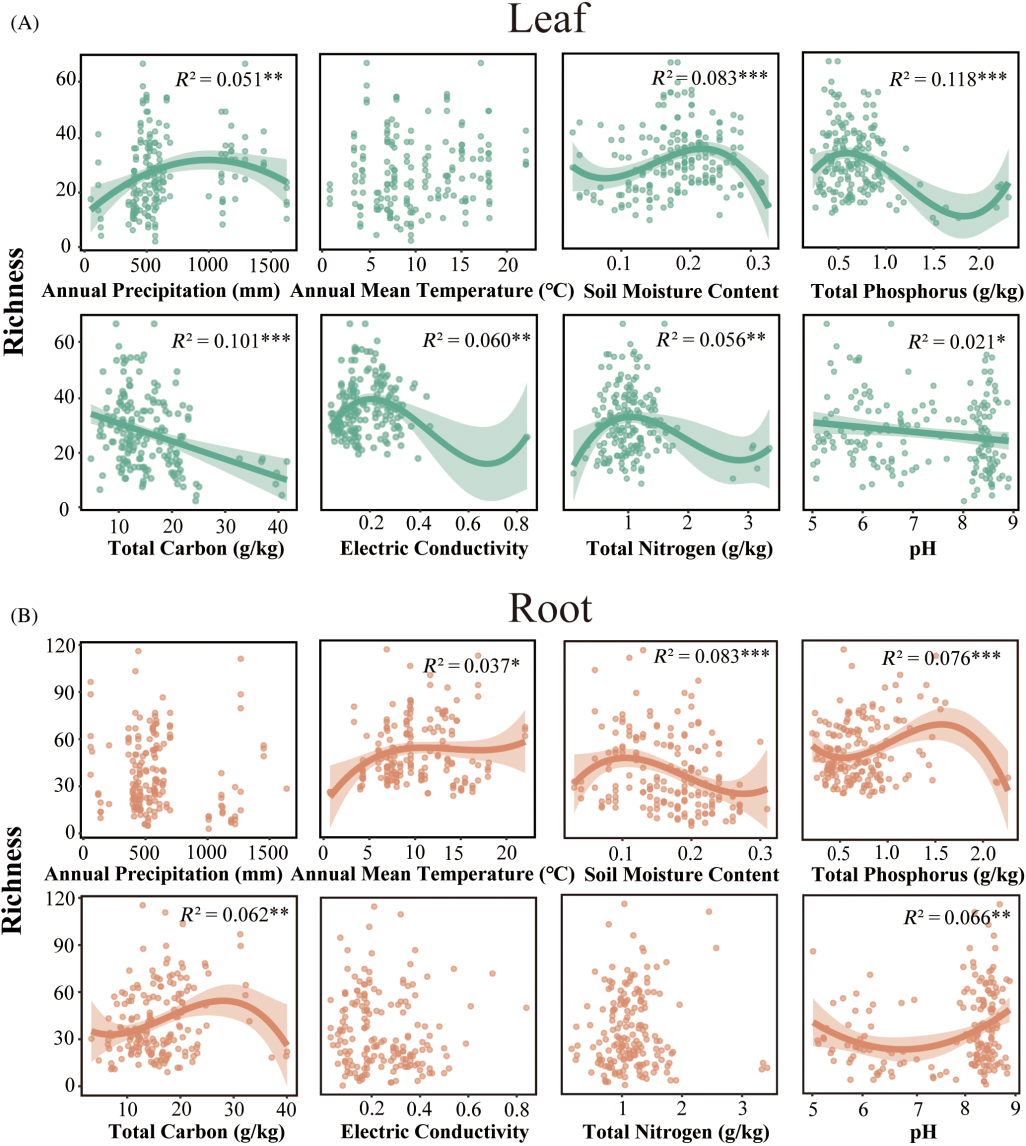
The leaf protist alpha diversity (A) and root protist alpha diversity (B) in relation to climatic factors and soil properties. The *R* ^2^ value, derived from ordinary least squares (OLS) regression, quantifies the percentage of variation in alpha diversity explained by the included predictor variables. Significant relationships are indicated by:*p* values <0.05*, <0.01** and <0.001***. Uncorrelated factors (including NO_3_^−^ −N, NH_4_^+^−xN, DOC, DON and EC) are not shown.

We assessed the correlations between the alpha diversity of protists in both leaf and root samples and various abiotic factors using the OLS regression analysis. The alpha diversity of leaf protists and TP exhibited the most pronounced correlation, followed by its correlations with soil attributes, namely TN, TC and SMC (Figure 3A). In root, the alpha diversity of protists exhibited the most significant correlation with SMC, followed by TP, soil pH and DOC (Figure 3B).

**FIGURE 4:**
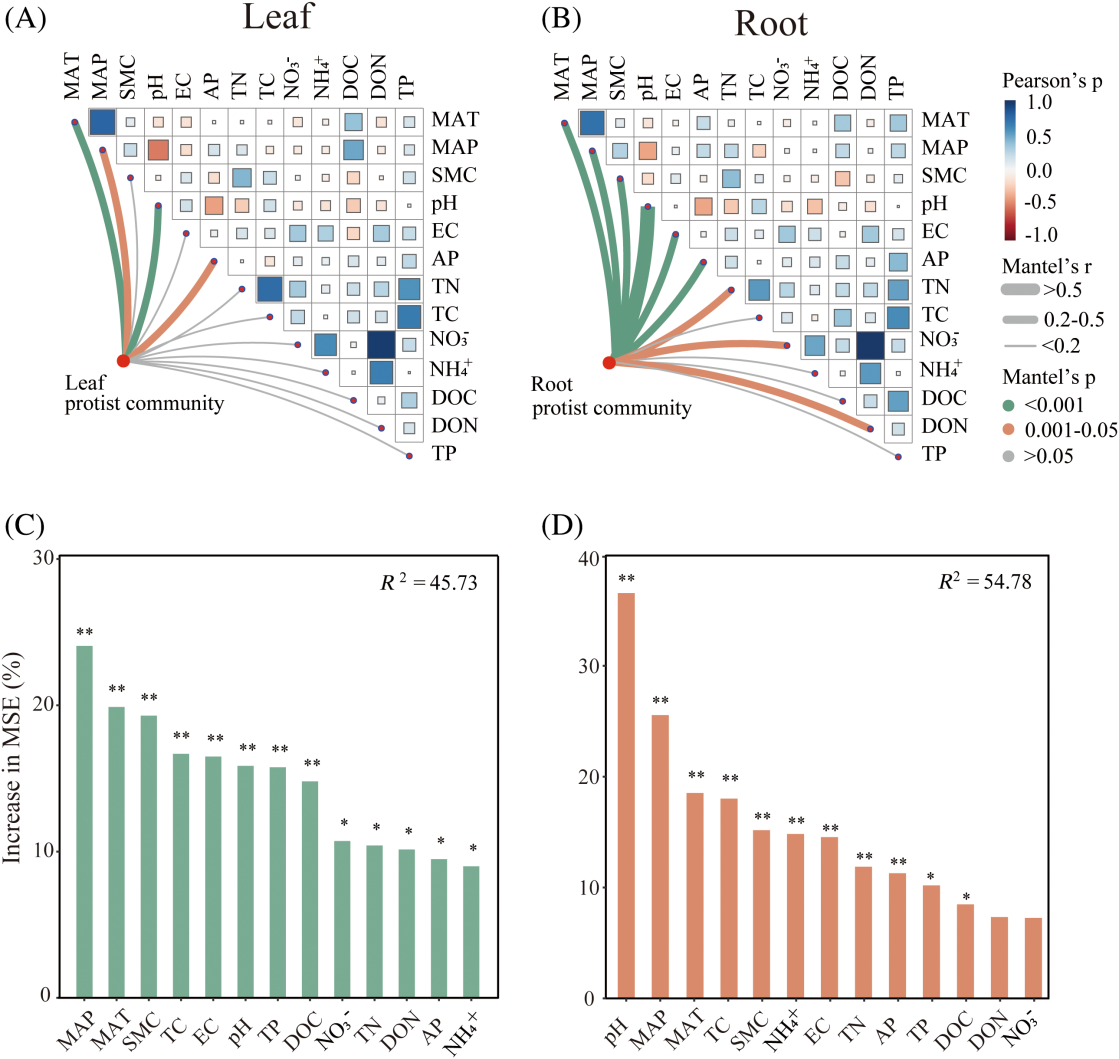
Mantel tests (A, B) were employed to unveil the relationships between protistan community composition, as measured by the Bray–Curtis dissimilarity, and various environmental factors. The relative importance of different factors in validating climatic and soil factors in predicting the community structure of leaf (C) and root (D) protists was determined by random forest modelling.

We identified the key factors structuring the compositions of protists in the leaf and root. The Mantel test revealed that the climate factors with the greatest predictive effect on the composition of protists in the leaf were MAP (*r* = 0.137, *p* < 0.001) and MAT (*r* = 0.088, *p* < 0.001) (Figure 4A). Soil pH had the most significant effect on the composition of protists in the root (*r* = 0.348, *p* < 0.001), followed by MAT (*r* = 0.210, *p* < 0.001) (Figure 4B). Results from random forest modelling indicated that MAP and soil pH were the most important predictors for the protistan community structures in the leaf and root, respectively (Figure 4C,D).

### Distribution of dominant protistan functional groups

**FIGURE 5:**
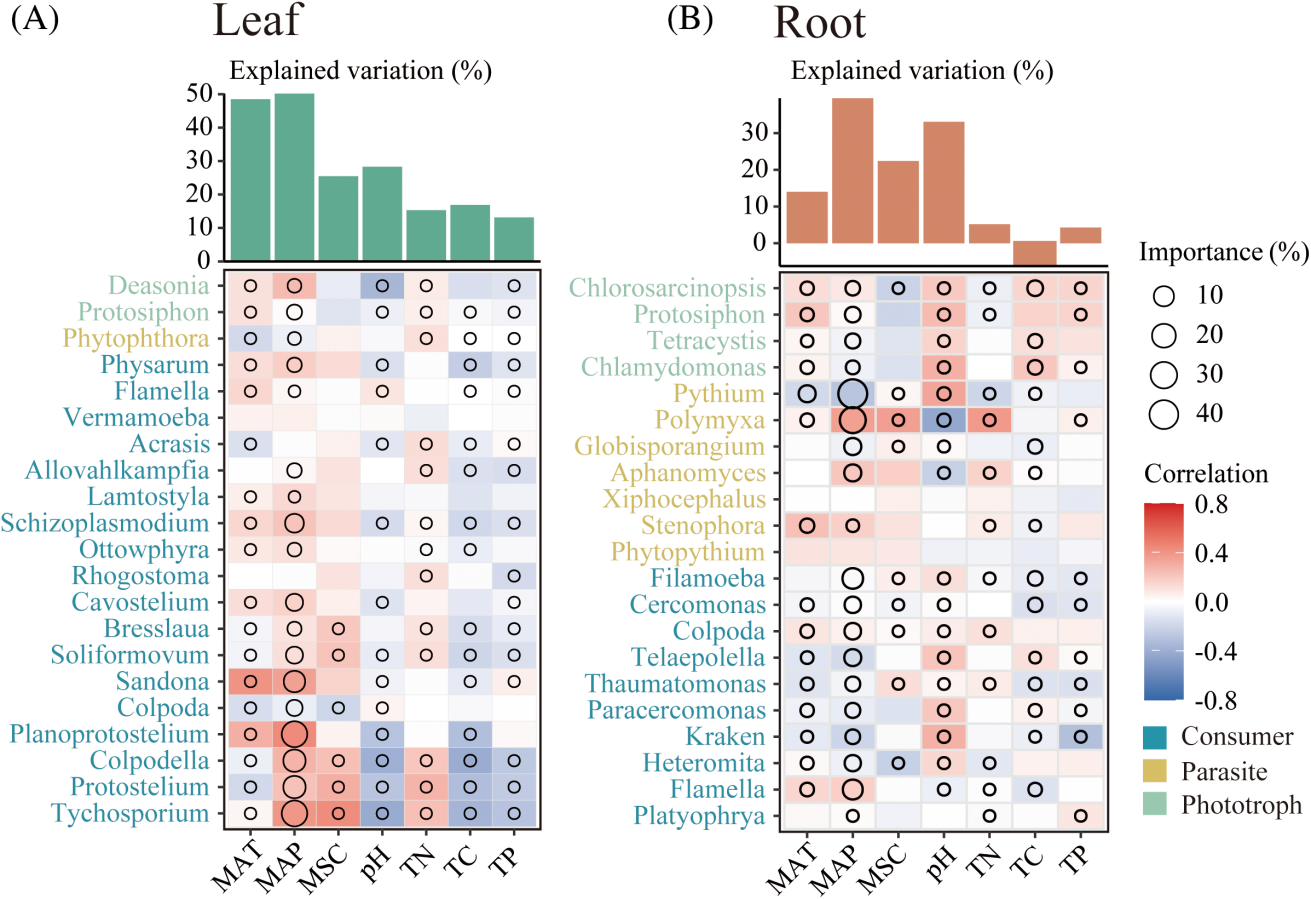
Associations between soil factors and climatic factors and the relative abundance of functional protist taxa (at the genus level) were estimated by Spearmans correlations (A, B). The size of the circles represents the significance of significant predictors (*p* < 0.05) for explaining changes in protist abundance through Random Forest modelling analyses. Different coloured genera represent different functional taxa.

To enhance our analysis of spatial distribution patterns among protistan functional groups, we selected genera with a relative abundance surpassing a 0.1% threshold. In the leaf, 98.40% of the genera were categorized as consumer protists, with *Tychosporium*, *Protostelium* and *Colpodella* being the predominant genera (Figure 5A). In the root, the protistan community composition was predominantly dominated by a greater number of parasitic protists (Figure 5B). Significant correlations were observed between the relative abundance of consumer genera in the leaf and various climatic factors, as determined through Spearman's rank correlation analysis (Figure 5A). Random forest models revealed that the relative abundance of predominant protist genera was most effectively predicted by MAP in both the leaf and root (Figure 5A,B). The relative abundance of protist functional groups was less influenced by edaphic factors such as SMC, pH, TP, TC and TN, compared to climatic variables in the root.

### Impact of climate and soil drivers on protistan community compositions

**FIGURE 6:**
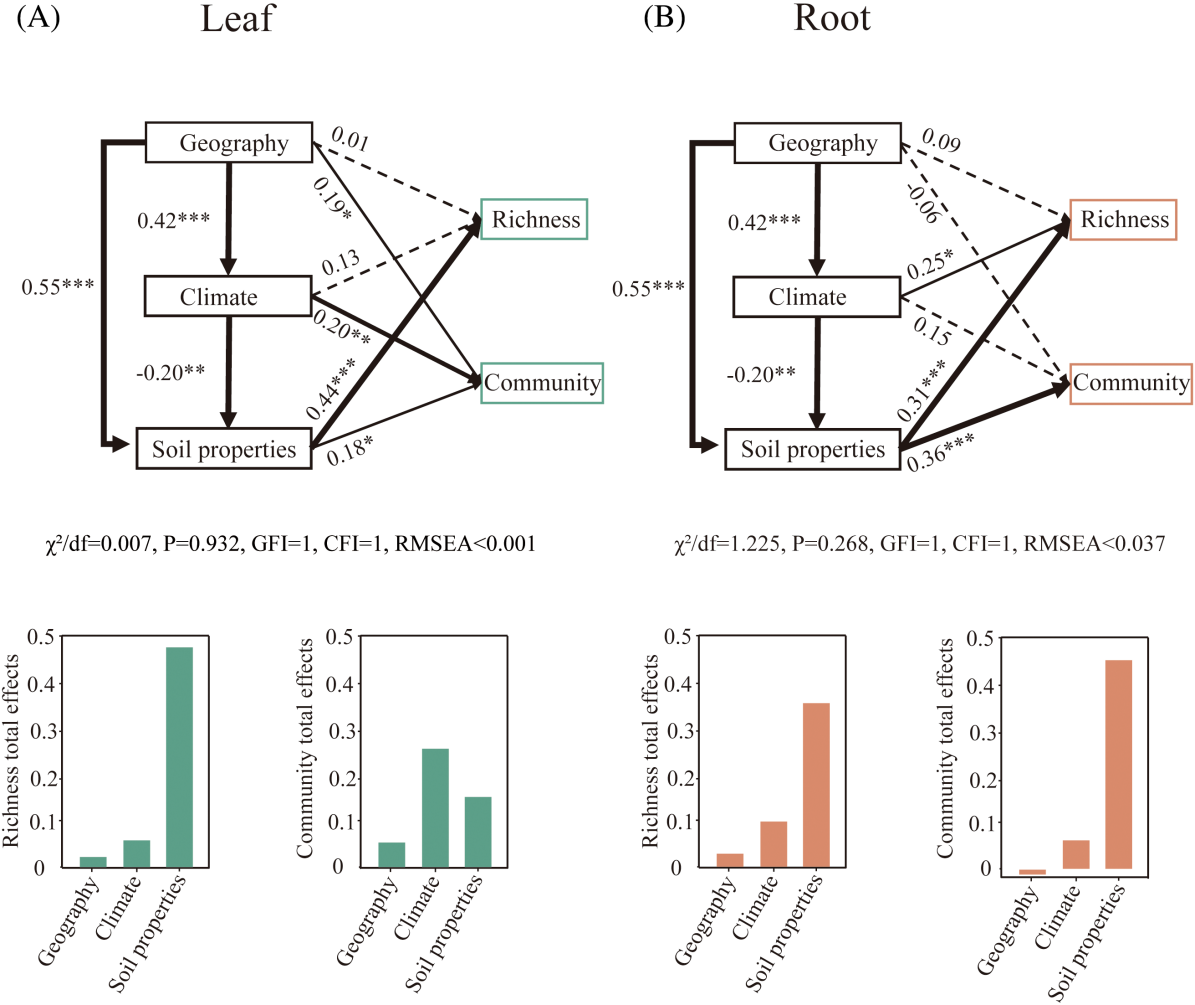
Structural equation models showing the direct and indirect effects of climatic and edaphic factors on the composition patterns of the leaf protists (A) and root protists (B). Edaphic factors (pH, EC, MSC, TN, TC, TP, DOC, DON, AP, NO_3_^−^−N, NH_4_^+^−N) and climatic factors (MAT, MAP) were represented by first axes of Principal Component Analysis (PCA1). Solid and dashed arrows indicate significant and non‐significant relationships, respectively. Numbers next to arrows are path coefficients, *p* values <0.05*, < 0.01** and <0.001***. The total standardized effects of multiple factors on the protistan richness and community compositions.

SEM was used to explore the causal links between the richness and community composition of protists with climatic variables, edaphic variables and geographic distances in both the leaf and root. Soil properties had a strong direct effect on the diversity of leaf protists (Figure 6A). Climatic factors significantly impacted the structure of the leaf protistan community through both direct and indirect effects. Simultaneously, soil properties and geographic distance exerted a direct influence on the leaf protistan communities (Figure 6A). Subsequently, we quantified the impact of abiotic factors on the diversity and community structure of protists in root. Soil properties significantly and strongly influenced root protistan species diversity through direct action. Climatic factors also contributed to the diversity of protists in the root, by acting both directly and indirectly (Figure 6B). Soil properties exerted a significant and direct effect on the community structure of protists in the root; neither climatic factors nor geographic distance had a direct effect (Figure 6B).

## DISCUSSION

Protists are crucial for promoting plant growth, suppressing pathogens and cycling nutrients (Xiong et al., 2020; Geisen et al., 2021; Guo et al., 2022). Diverse compartments of the plant exhibit distinct structural features, each subjected to a unique set of environmental conditions, thereby potentially attracting a varied spectrum of microbial colonizers (Fitzpatrick et al., 2020). In the leaf, protists showed lower richness compared with root compartments, supporting previous perceptions that diversity is rapidly lost from root to leaf due to host‐specific factors (Trivedi et al., 2020). Our study, which compared protists in the compartments of sorghum plants, revealed a marked distinction between the protist communities associated with the leaf and root. Evosea, Cercozoa and Ciliophora were predominantly observed in the leaf, whereas Endomyxa, Oomycota and Cercozoa were primarily found in the root. Previous studies have demonstrated marked disparities in the species composition and community architecture of bacteria and fungi across various plant compartments, including sugarcane (Teheran‐Sierra et al., 2021), tomato (De Souza et al., 2016) and sorghum (Sun, Jiao, Chen, Wu, et al., 2021). Marked disparities in the diversity and community structure of protists in sorghum's leaf and root mirror those documented in bacterial and fungal communities, suggesting a predominant effect of plant compartments in governing their associated microorganisms (Trivedi et al., 2020).

We found that 65% of the leaf protists, and 25% of the root protists, remained taxonomically unclassified. This indicated that the plant‐associated protistan community harbours a vast reservoir of novel taxa yet to be discovered. To date, most studies of plant‐associated protists have relied on microscopy and direct counting, quantitative PCR or amplicon sequencing. However, the wide diversity of eukaryotic microbial populations presents ongoing challenges in identifying microbial eukaryotes using high‐throughput sequencing (Nguyen et al., 2023). Additionally, the use of poorly designed primers in PCR analyses can lead to non‐specific amplification and the generation of non‐target sequences. Designing accurate primers for the major group of plant‐associated protists is not so easy, and serious primer bias has been found in previous surveys of protists (Lentendu et al., 2014). It is also important to note that we did not use peptide‐nucleic acid (PNA) or locked nucleic acid (LNA) to inhibit plant‐origin DNA during PCR amplification, which may have resulted in a majority of sequences being plant‐origin.

For the protist functional groups, a significant divergence exists between the leaf and root. Our study revealed that consumer taxa predominate in the leaf protistan community (Figures 2 and 5). Given the leaf openness, and the presence of microbial communities as food sources, it is not surprising that protistan consumers are predominant (Gong & Xin, 2021; Koskella, 2020; Li et al., 2022; Mechan‐Llontop et al., 2023; Sohrabi et al., 2023; Vacher et al., 2016). Protistan consumers occupy diverse ecological niches and utilize a range of food sources, suggesting that they are better able to adapt to environmental changes (Geisen et al., 2018). Protist consumers tend to better survive in nutrient‐poor leaf with variable environmental conditions compared with phototrophs and parasites. Through predation on microorganisms, protists facilitate nutrient cycling by releasing inorganic and organic nutrients (Gao et al., 2019; Geisen et al., 2021; Guo et al., 2022), which can subsequently be utilized by plants to support growth and development. As significant microbial predators, protists also directly engulf bacterial and fungal pathogens. The predation of protists can result in the demise of a diverse range of bacterial and fungal strains (Chakraborty et al., 1983; Dumack et al., 2016). In addition, when plants are attacked by pathogens or pests, protists may respond by recruiting antibiotic producers to synthesize antimicrobials that inhibit the pathogens or pests, thereby protecting the plant (Liu et al., 2019). In vitro investigations have demonstrated that predation by foliar protists, belonging to the Cercozoa and Cercomonas taxa, significantly alters the taxonomic structure and metabolic profiles of bacterial communities in leaves (Flues et al., 2017). Furthermore, it has been reported that predatory protists play a pivotal role in pathogen suppression, indicating the potential of leaf protists as biological agents in the management and control of plant pathogens (Guo et al., 2021; Ren et al., 2023). The dominance of protist consumers may indicate that they can regulate bacterial and fungal communities, thereby influencing ecosystem processes such as nutrient cycling and pathogen control.

According to our large‐scale field surveys, sorghum root endosperms selectively recruited a higher number of parasites (Figures 2 and 5). The root may be richer in nutrients and water, leading to the recruitment of more parasitic protists within the root (Bulgarelli et al., 2012; Molefe et al., 2023). Compared with the leaf, the root is more stable and less susceptible to external environmental influences, facilitating the long‐term colonization of protists (Lundberg et al., 2012; Molefe et al., 2023; Salem et al., 2022). Additionally, the relatively weak defence response of roots allows numerous microorganisms to invade and colonize root cells, which also promotes the survival and reproduction of protistan parasites (Bulgarelli et al., 2012; Mendes et al., 2013; Yue et al., 2023). Within the sorghum root microbiome, our investigations revealed a predominance of parasitic protists belonging to Endomyxa, Oomycota and Apicomplexa. Members of Endomyxa, including the genus *Polymyxa*, are widely distributed across diverse plant hosts, and act as viral vectors in cereal crops (Kanyuka et al., 2003). The oomycete genus *Pythium* is well known for its role in suppressing disease (Savory et al., 2015) and is prevalent in the sorghum root microbiome in our study. While a small group of oomycetes are known to inflict disease upon plants, non‐pathogenic oomycetes are often unnoticed, yet they can exert a beneficial influence on plant growth (Benhamou et al., 2012; Sapp et al., 2018). Root endophytic protists are more closely linked to the immune mechanisms of the plant host, implying greater pathogen resistance. In addition, symbiotic interactions between root endophytic protists and plant hosts potentially influence the transfer of phytohormones and nutrients to plant tissues.

We explored the influence of soil properties and climatic factors in shaping the diversity and community composition of protists in the leaf and root. The SEM analysis further elucidated the direct and indirect relationships between climatic factors, soil properties and geographic distances, and protist diversity and community structure (Figure 6). Surprisingly, the diversity of both leaf and root protists, showed stronger correlations with soil properties and was less influenced by climatic factors. Additionally, we identified climatic factors as the primary predictors of the community structure of leaf protists. Soil properties play an important role in shaping the community structure of protists in the root. Our further research found that MAP is the primary determinant of leaf protist community composition. Recent studies have identified moisture as a key driver of protist diversity and community, highlighting the crucial role of water availability for protist survival, dispersal and reproduction (Oliverio et al., 2020). Soil pH was identified as the strongest predictor of protist community composition in the root (Figure 4). The unique environmental conditions within the root result in protist community structure being primarily governed by soil physicochemical properties. Leaf protists are more sensitive and responsive to future climate change, and below‐ground soil properties more strongly regulate root protists. The results will also help predict the responses of protistan communities to climate change and environmental changes from anthropogenic disturbances, as well as aid in the management and utilization of protists in agro‐ecosystems.

## CONCLUSIONS

In summary, we conducted a comprehensive investigation of the diversity and community structure of leaf and root protists, as well as their driving factors, through a large‐scale, multi‐site field experiment. We provide new evidence that leaves are dominated by protist consumers, while roots are dominated by protist parasites, suggesting different ecosystem functions of protists in these contrasting habitats. Protistan communities in leaves are mainly influenced by MAP, whereas soil pH is the dominant predictor of root protists. This suggests that changes in precipitation patterns under future climatic scenarios and soil pH alterations due to long‐term fertilization would significantly impact leaf and root protists and their ecosystem functions, with unknown consequences for plant health and performance. These findings advance our knowledge of protistan communities associated with sorghum crop plants at a national scale and highlight the necessity of future studies to unravel the actual functional roles played by protists in plant habitats.

## AUTHOR CONTRIBUTIONS

**Peng He:** Investigation; methodology; writing – review and editing; writing – original draft; visualization. **Anqi Sun:** Conceptualization; investigation; writing – review and editing; visualization; methodology. **Xiaoyan Jiao:** Funding acquisition; conceptualization; writing – review and editing; data curation; resources. **Peixin Ren:** Writing – review and editing. **Fangfang Li:** Writing – review and editing; supervision. **Bingxue Wu:** Investigation; methodology; writing – review and editing. **Ji‐Zheng He:** Conceptualization; project administration; writing – review and editing; supervision. **Hang‐Wei Hu:** Writing – review and editing; writing – original draft; conceptualization; project administration; supervision; resources.

## CONFLICT OF INTEREST STATEMENT

The authors declare no conflicts of interest.

## Supporting information


**Data S1.** Supporting Information.

## Data Availability

We have deposited all raw sequences in the NCBI Sequence Read Archive (SRA) under the accession number PRJNA1154307.
