# Supplementary material for: National‐scale distribution of protists associated with sorghum leaves and roots
Source: Environ Microbiol Rep. 2024 Oct 1;16(5):e70024. doi: 10.1111/1758-2229.70024 (PMC11443160; doi:10.1111/1758-2229.70024)
Supplement: Supplementary file 1 — Data S1. Supporting Information. [file EMI4-16-e70024-s001.docx]

**Supplementary Materials**

*Title page*

**National-scale distribution of protists associated with sorghum leaves and roots**

Peng He ^1^, Anqi Sun ^2^, Xiaoyan Jiao ^3^, Peixin Ren ^1^, Fangfang Li ^1^, Bingxue Wu ^1^, Ji-Zheng He ^4^, Hang-Wei Hu ^4,^*

*^1^ Key Laboratory for Humid Subtropical Eco-geographical Processes of the Ministry of Education, School of Geographical Sciences, Fujian Normal University, Fuzhou, China*

*^2^ Key Laboratory of Urban Environment and Health, Ningbo Urban Environment Observation and Research Station, Institute of Urban Environment, Chinese Academy of Sciences, Xiamen 361021, China*

*^3^ College of Resources and Environment, Shanxi Agricultural University, Taiyuan 030031, China*

*^4^ School of Agriculture, Food and Ecosystem Sciences, Faculty of Science, The University of Melbourne, Parkville, Victoria, 3010, Australia*

For correspondence: Hang-Wei Hu,

Email: [hang-wei.hu@unimelb.edu.au](mailto:hang-wei.hu@unimelb.edu.au).

|  |
| --- |

| 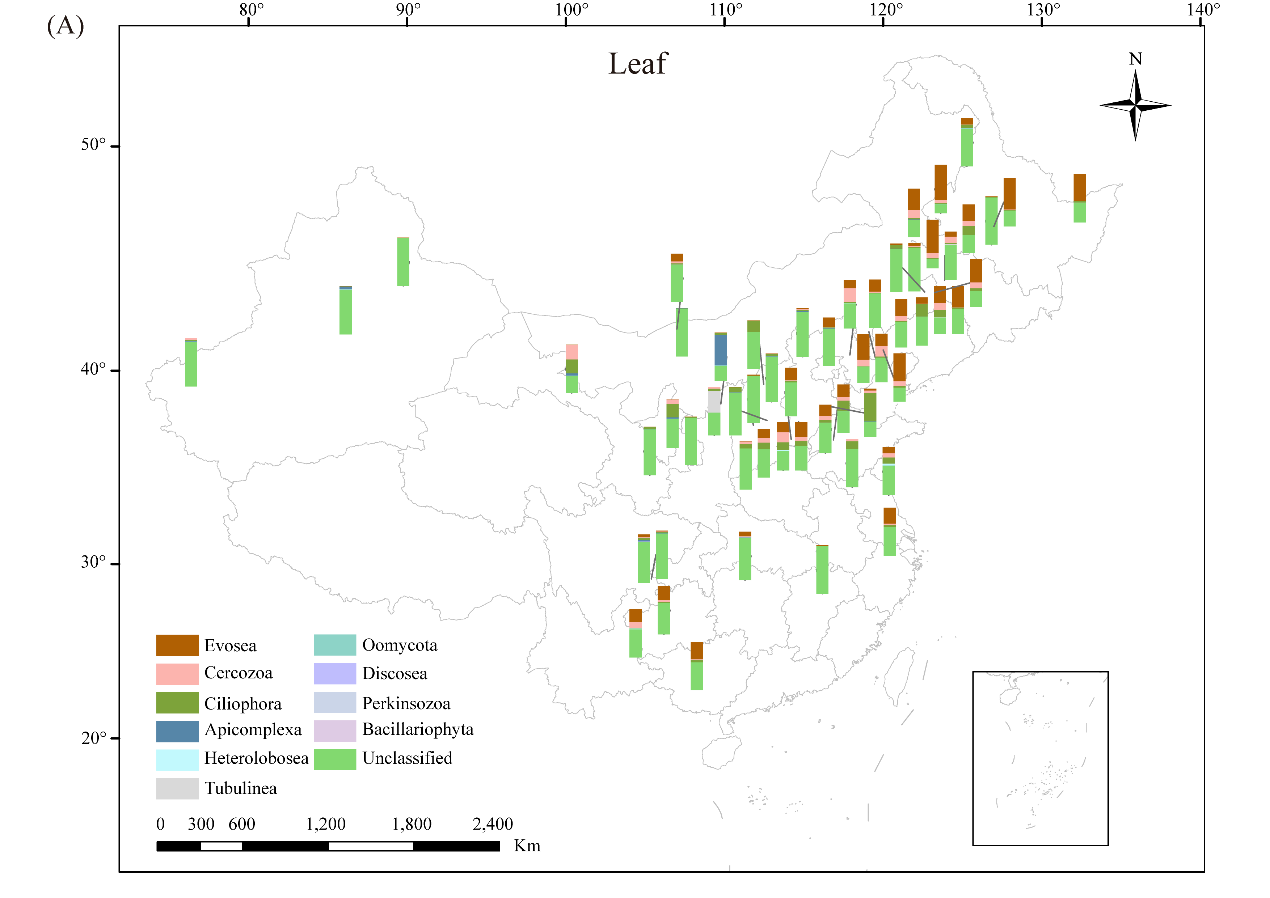   \|  \| \| --- \| \| 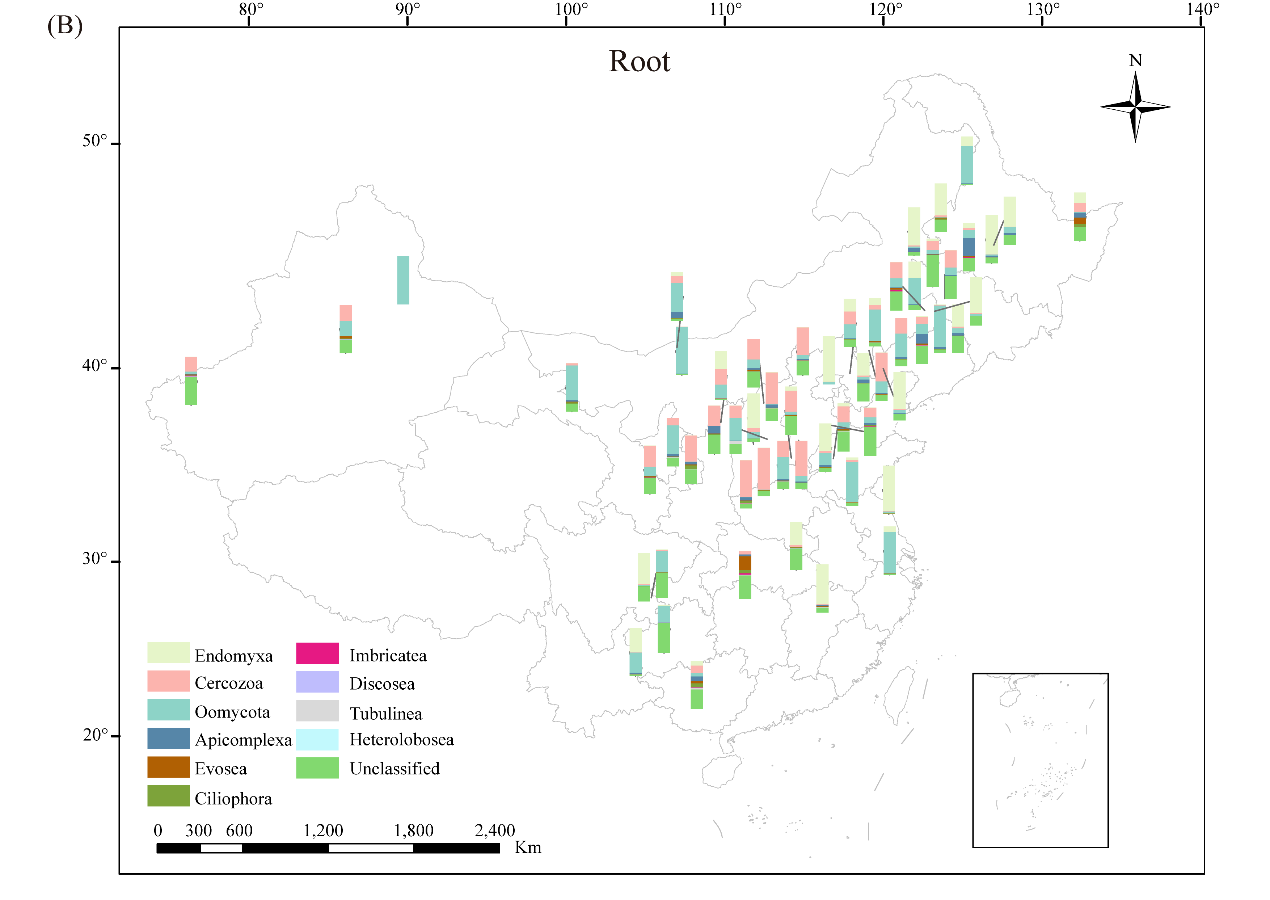 \| \| **Fig. S1.** The map shows the composition of protists in the sorghum leaf (A) and root (B) at the 57 sampling sites. \|   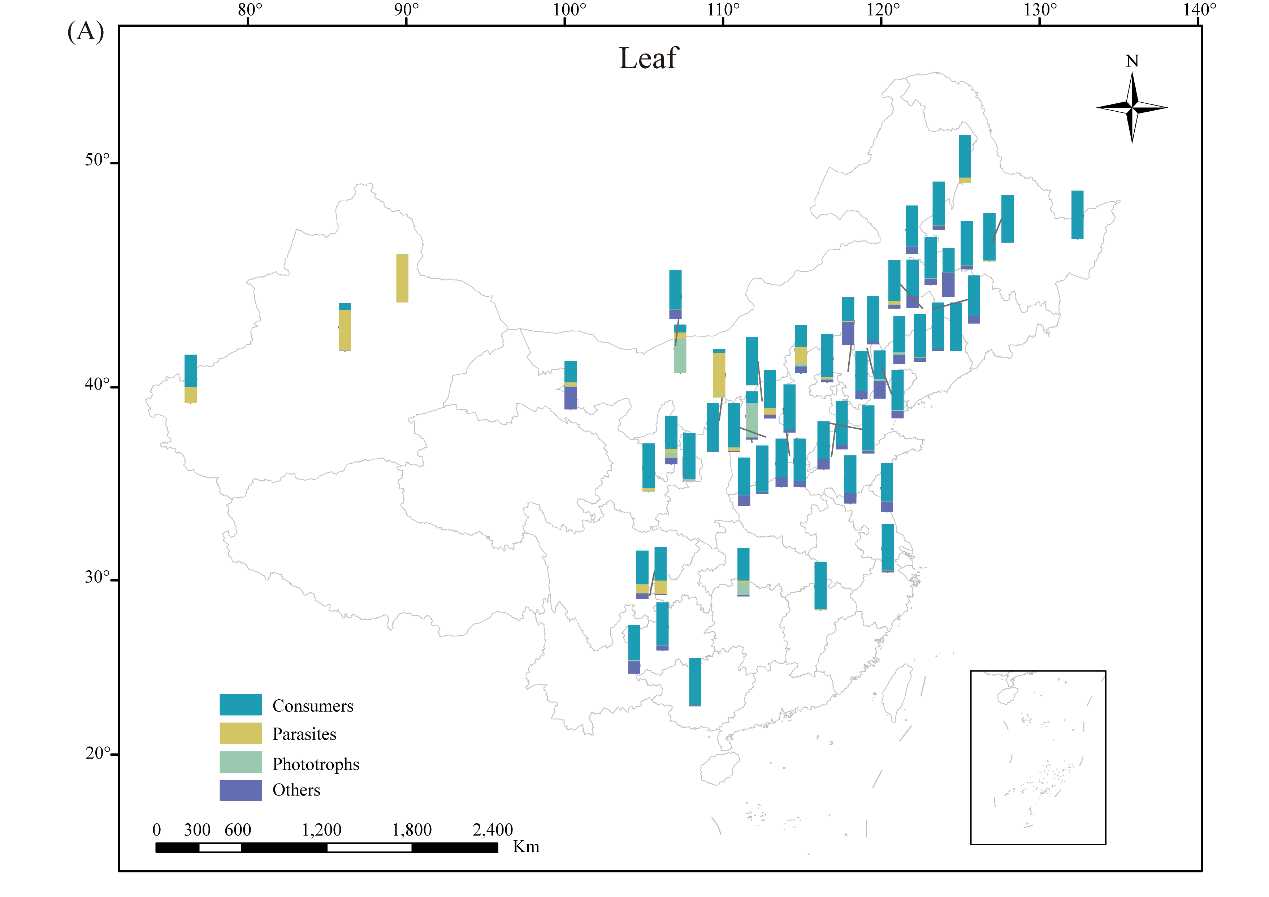   \| 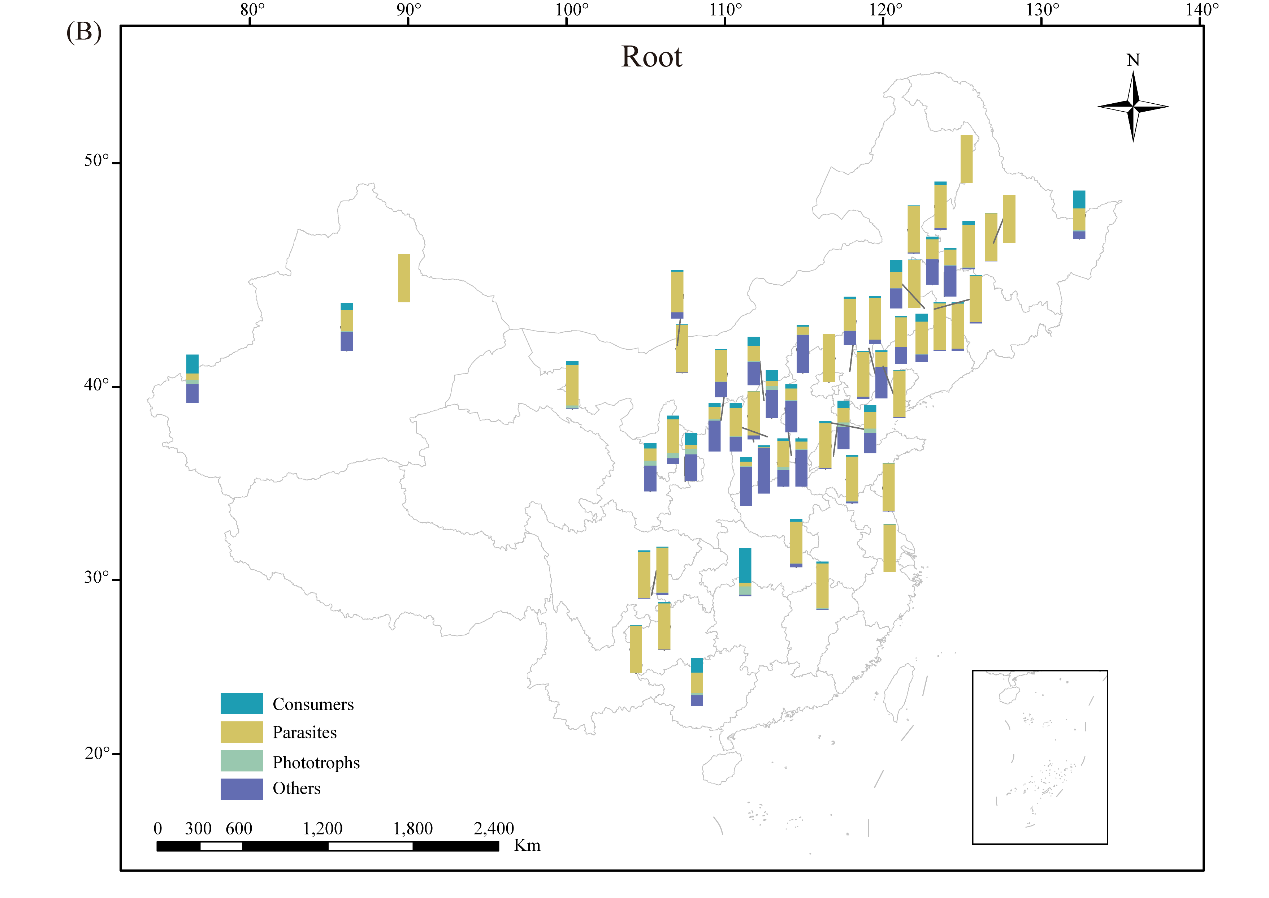 \| \| --- \| \| **Fig. S2.** The map shows the composition of functional taxa of protists in sorghum leaf (A) and root (B) at the 57 sampling sites. \| |
| --- | --- | --- | --- | --- | --- |

| **Table S1.** Location and site information at 57 sampling sites. | | | | | | | | | | | | | | | |
| --- | --- | --- | --- | --- | --- | --- | --- | --- | --- | --- | --- | --- | --- | --- | --- |
| Site | longitude | Latitude | MAT(°C) | MAP(mm) | MSC(%) | pH | EC | AP(mg/kg) | TN(g/kg) | TC(g/kg) | NO_3_^–^-N(mg/kg) | NH_4_^+^-N(mg/kg) | DOC(mg/kg) | DON(mg/kg) | TP(g/kg) |
| 1 | 120.00 | 41.00 | 7.46 | 534.00 | 0.23 | 8.43 | 0.33 | 11.33 | 1.07 | 11.95 | 7.94 | 3.00 | 25.55 | 3.77 | 0.38 |
| 2 | 123.63 | 47.21 | 4.30 | 425.00 | 0.24 | 8.17 | 0.41 | 13.60 | 1.63 | 23.46 | 14.08 | 3.08 | 24.13 | 7.90 | 0.70 |
| 3 | 126.97 | 46.63 | 3.33 | 536.00 | 0.22 | 5.59 | 0.08 | 9.55 | 1.72 | 20.96 | 14.19 | 3.65 | 30.87 | 15.34 | 0.50 |
| 4 | 132.39 | 46.81 | 3.33 | 536.00 | 0.09 | 5.96 | 0.40 | 57.96 | 1.27 | 11.59 | 18.94 | 3.73 | 27.20 | 19.08 | 0.90 |
| 5 | 125.28 | 49.18 | 0.69 | 496.00 | 0.26 | 6.07 | 0.15 | 16.04 | 1.90 | 19.47 | 9.89 | 6.29 | 35.05 | 9.76 | 0.84 |
| 6 | 119.10 | 41.86 | 7.72 | 410.00 | 0.16 | 8.28 | 0.42 | 12.02 | 1.19 | 12.50 | 12.97 | 2.87 | 33.06 | 10.59 | 0.67 |
| 7 | 123.23 | 43.68 | 6.95 | 462.00 | 0.24 | 8.54 | 0.36 | 6.88 | 1.22 | 16.82 | 8.65 | 2.04 | 42.33 | 10.98 | 0.56 |
| 8 | 121.94 | 46.17 | 4.21 | 410.00 | 0.22 | 6.67 | 0.31 | 4.09 | 1.85 | 17.74 | 27.68 | 4.19 | 19.90 | 27.81 | 0.37 |
| 9 | 122.62 | 43.72 | 6.90 | 444.00 | 0.13 | 8.64 | 0.21 | 5.13 | 1.04 | 12.91 | 7.45 | 2.49 | 62.67 | 8.83 | 0.54 |
| 10 | 107.00 | 42.00 | 6.93 | 123.00 | 0.13 | 8.37 | 0.33 | 8.50 | 0.99 | 10.70 | 10.38 | 2.72 | 35.00 | 7.98 | 0.39 |
| 11 | 107.31 | 40.70 | 7.99 | 142.00 | 0.15 | 8.76 | 0.18 | 21.00 | 1.03 | 23.08 | 7.59 | 2.71 | 36.45 | 7.97 | 0.92 |
| 12 | 121.14 | 41.12 | 9.54 | 578.00 | 0.13 | 5.87 | 0.29 | 16.59 | 0.95 | 10.51 | 2.53 | 16.88 | 60.83 | 9.30 | 0.42 |
| 13 | 114.66 | 37.49 | 13.45 | 509.00 | 0.19 | 8.40 | 0.17 | 2.37 | 1.54 | 13.83 | 10.95 | 5.74 | 47.49 | 9.15 | 0.40 |
| 14 | 114.92 | 40.68 | 9.08 | 366.00 | 0.12 | 8.45 | 0.17 | 30.78 | 1.63 | 22.53 | 6.66 | 2.94 | 41.80 | 7.36 | 0.35 |
| 15 | 117.90 | 40.77 | 9.09 | 505.00 | 0.20 | 8.25 | 0.09 | 10.59 | 1.16 | 9.14 | 6.54 | 1.87 | 27.51 | 9.68 | 0.36 |
| 16 | 118.75 | 39.42 | 11.08 | 614.00 | 0.18 | 6.61 | 0.12 | 60.11 | 1.35 | 10.74 | 14.26 | 3.55 | 49.57 | 14.25 | 1.19 |
| 17 | 116.76 | 38.24 | 13.04 | 471.00 | 0.14 | 8.71 | 0.14 | 7.38 | 1.00 | 17.90 | 7.10 | 1.92 | 26.78 | 6.32 | 0.93 |
| 18 | 116.86 | 36.57 | 13.32 | 698.00 | 0.20 | 8.84 | 0.19 | 8.93 | 0.80 | 17.98 | 3.10 | 2.25 | 40.44 | 5.77 | 0.75 |
| 19 | 116.36 | 35.90 | 13.56 | 640.00 | 0.16 | 5.73 | 0.18 | 30.61 | 1.32 | 12.34 | 25.62 | 69.84 | 44.40 | 26.02 | 0.90 |
| 20 | 118.04 | 34.13 | 14.58 | 699.00 | 0.19 | 7.92 | 0.09 | 11.29 | 1.53 | 16.32 | 17.85 | 2.66 | 49.07 | 16.60 | 1.17 |
| 21 | 112.47 | 39.34 | 7.25 | 401.00 | 0.04 | 8.68 | 0.11 | 16.89 | 0.87 | 20.39 | 20.01 | 1.79 | 100.36 | 14.75 | 0.62 |
| 22 | 112.95 | 38.48 | 9.48 | 421.00 | 0.07 | 8.51 | 0.17 | 5.41 | 0.84 | 20.06 | 2.88 | 3.34 | 45.95 | 5.80 | 0.70 |
| 23 | 119.48 | 42.05 | 7.30 | 413.00 | 0.10 | 8.51 | 0.16 | 3.88 | 0.76 | 11.46 | 6.89 | 2.94 | 33.42 | 4.01 | 0.85 |
| 24 | 111.69 | 35.27 | 13.02 | 560.00 | 0.15 | 8.36 | 0.37 | 6.63 | 1.05 | 15.64 | 4.90 | 1.84 | 26.74 | 5.75 | 0.60 |
| 25 | 112.68 | 37.55 | 10.05 | 432.00 | 0.05 | 8.36 | 0.16 | 3.14 | 0.93 | 20.47 | 9.53 | 2.66 | 38.17 | 9.78 | 0.83 |
| 26 | 111.83 | 37.29 | 10.70 | 452.00 | 0.08 | 8.19 | 0.24 | 19.71 | 0.62 | 15.47 | 34.16 | 34.60 | 45.83 | 25.37 | 0.84 |
| 27 | 112.48 | 34.64 | 15.10 | 609.00 | 0.18 | 8.15 | 0.19 | 9.78 | 1.44 | 19.98 | 28.57 | 2.09 | 36.24 | 22.75 | 0.86 |
| 28 | 114.20 | 36.60 | 12.51 | 590.00 | 0.24 | 8.18 | 0.35 | 13.93 | 1.55 | 19.91 | 16.61 | 1.98 | 31.95 | 21.29 | 0.96 |
| 29 | 113.70 | 35.00 | 14.04 | 570.00 | 0.09 | 8.25 | 0.22 | 12.35 | 0.88 | 17.43 | 61.21 | 32.27 | 42.74 | 55.46 | 0.69 |
| 30 | 107.50 | 35.28 | 9.44 | 588.00 | 0.09 | 7.98 | 0.14 | 8.45 | 1.25 | 14.89 | 59.81 | 3.15 | 57.09 | 66.08 | 0.83 |
| 31 | 100.37 | 38.92 | 6.92 | 210.00 | 0.18 | 8.54 | 0.09 | 25.67 | 1.09 | 20.53 | 8.23 | 3.65 | 43.82 | 9.40 | 1.00 |
| 32 | 105.30 | 34.75 | 9.53 | 579.00 | 0.10 | 8.47 | 0.19 | 17.98 | 1.14 | 25.25 | 9.19 | 3.29 | 58.87 | 8.99 | 1.56 |
| 33 | 123.79 | 42.25 | 7.75 | 648.00 | 0.10 | 5.40 | 0.03 | 8.81 | 0.93 | 9.63 | 14.08 | 3.39 | 25.84 | 8.63 | 0.41 |
| 34 | 109.77 | 38.38 | 8.39 | 399.00 | 0.06 | 8.20 | 0.21 | 10.45 | 0.26 | 7.28 | 33.66 | 2.67 | 37.68 | 35.95 | 0.51 |
| 35 | 109.36 | 36.80 | 9.69 | 495.00 | 0.09 | 8.14 | 1.36 | 16.37 | 0.69 | 20.37 | 41.21 | 32.21 | 50.73 | 47.36 | 0.52 |
| 36 | 76.38 | 39.23 | 12.05 | 74.00 | 0.09 | 8.14 | 1.36 | 16.37 | 0.69 | 20.37 | 41.21 | 32.21 | 50.73 | 47.36 | 0.69 |
| 37 | 89.75 | 43.99 | 5.76 | 145.00 | 0.18 | 8.23 | 0.27 | 7.21 | 2.01 | 33.67 | 31.74 | 2.75 | 57.15 | 31.65 | 1.67 |
| 38 | 106.73 | 36.17 | 7.56 | 456.00 | 0.14 | 8.62 | 0.50 | 11.74 | 0.52 | 20.43 | 11.41 | 1.87 | 51.60 | 11.84 | 0.90 |
| 39 | 116.58 | 40.24 | 11.41 | 520.00 | 0.25 | 8.26 | 0.32 | 5.62 | 0.67 | 6.86 | 2.68 | 3.50 | 24.93 | 3.46 | 0.56 |
| 40 | 105.38 | 29.18 | 17.99 | 1110.00 | 0.20 | 5.11 | 0.11 | 14.42 | 0.93 | 10.82 | 25.16 | 1.97 | 44.27 | 17.97 | 0.40 |
| 41 | 104.91 | 28.97 | 18.00 | 1143.00 | 0.21 | 6.61 | 0.16 | 18.03 | 1.65 | 21.24 | 73.65 | 2.33 | 41.94 | 11.89 | 0.79 |
| 42 | 124.03 | 43.18 | 6.98 | 574.00 | 0.19 | 6.43 | 0.44 | 13.61 | 1.53 | 15.54 | 118.98 | 38.73 | 25.65 | 161.37 | 0.60 |
| 43 | 106.18 | 26.07 | 15.06 | 1189.00 | 0.21 | 6.33 | 0.15 | 29.43 | 0.95 | 10.46 | 13.50 | 2.36 | 50.20 | 13.63 | 0.35 |
| 44 | 120.41 | 30.44 | 16.30 | 1220.00 | 0.25 | 7.75 | 0.39 | 26.99 | 0.72 | 7.24 | 4.23 | 2.15 | 33.44 | 5.11 | 0.74 |
| 45 | 104.39 | 24.75 | 15.48 | 1119.00 | 0.31 | 6.03 | 0.44 | 17.50 | 3.42 | 39.88 | 75.34 | 5.88 | 81.64 | 74.64 | 2.14 |
| 46 | 120.35 | 33.70 | 14.19 | 1008.00 | 0.23 | 8.31 | 0.11 | 10.66 | 0.96 | 16.38 | 37.11 | 2.07 | 36.73 | 32.69 | 0.78 |
| 47 | 108.25 | 22.85 | 22.06 | 1449.00 | 0.22 | 6.64 | 0.31 | 12.53 | 1.04 | 10.90 | 2.23 | 4.44 | 42.44 | 4.59 | 0.54 |
| 48 | 116.17 | 28.36 | 18.08 | 1633.00 | 0.14 | 4.84 | 0.04 | 26.60 | 1.20 | 11.72 | 6.54 | 4.55 | 174.84 | 19.65 | 0.71 |
| 49 | 111.29 | 29.11 | 17.15 | 1300.00 | 0.18 | 5.11 | 0.14 | 9.97 | 1.82 | 16.70 | 4.55 | 2.83 | 56.13 | 8.37 | 0.47 |
| 50 | 114.52 | 30.70 | 16.91 | 1265.00 | 0.12 | 8.03 | 0.38 | 47.88 | 2.57 | 17.30 | 48.98 | 1.89 | 55.15 | 54.11 | 1.41 |
| 51 | 122.80 | 45.62 | 5.81 | 405.00 | 0.17 | 6.73 | 0.11 | 13.14 | 1.02 | 11.22 | 48.56 | 3.09 | 19.22 | 55.88 | 0.30 |
| 52 | 123.57 | 41.82 | 7.95 | 671.00 | 0.18 | 5.88 | 0.24 | 19.24 | 1.02 | 11.99 | 5.51 | 2.59 | 32.17 | 6.25 | 0.77 |
| 53 | 86.12 | 41.74 | 11.66 | 62.00 | 0.21 | 8.41 | 0.07 | 34.04 | 1.35 | 32.96 | 7.79 | 2.27 | 52.88 | 10.40 | 1.33 |
| 54 | 125.30 | 45.48 | 4.70 | 480.00 | 0.16 | 5.19 | 0.29 | 32.59 | 1.34 | 10.94 | 25.07 | 11.63 | 33.49 | 44.13 | 0.47 |
| 55 | 123.87 | 44.26 | 6.00 | 457.00 | 0.19 | 8.54 | 0.06 | 7.12 | 1.00 | 13.60 | 8.55 | 3.10 | 31.23 | 8.24 | 0.41 |
| 56 | 123.11 | 44.79 | 6.28 | 427.00 | 0.15 | 8.66 | 0.06 | 8.30 | 0.60 | 14.07 | 14.10 | 3.46 | 29.69 | 8.62 | 0.26 |
| 57 | 126.83 | 45.84 | 4.01 | 523.00 | 0.15 | 6.45 | 0.14 | 12.24 | 1.34 | 15.17 | 19.02 | 1.94 | 36.12 | 24.17 | 0.43 |
